# The effect of high-intensity resistance exercise on lumbar musculature in patients with low back pain: a preliminary study

**DOI:** 10.1186/s12891-019-2658-1

**Published:** 2019-06-18

**Authors:** David B. Berry, Jennifer Padwal, Seth Johnson, Erin K. Englund, Samuel R. Ward, Bahar Shahidi

**Affiliations:** 1 Departments of Bioengineering, University of California San Diego, La Jolla, California USA; 2 Departments of Nanoengineering, University of California San Diego, La Jolla, California USA; 3 Departments of Medicine, University of California San Diego, La Jolla, California USA; 4 Departments of Orthopaedic Surgery, University of California San Diego, La Jolla, California USA; 5 Departments of Radiology, University of California San Diego, La Jolla, California USA

**Keywords:** Low back pain, MRI, Lumbar muscle, Resistance based exercise, Rehabilitation

## Abstract

**Background:**

Muscle atrophy and fatty infiltration of the lumbar extensors is associated with LBP. Exercise-based rehabilitation targets strengthening these muscles, but few studies show consistent changes in muscle quality with standard-of-care rehabilitation. The goal of this study was to assess the effect of high-intensity resistance exercise on lumbar extensor muscle size (cross sectional area) and quality (fat fraction) in individuals with low back pain (LBP).

**Methods:**

Fourteen patients with LBP were recruited from a local rehabilitation clinic. Patients underwent MRI scanning before and after a standardized 10-week high-intensity machine-based, resistance exercise program. Patient pain, disability, anxiety/depression, satisfaction, strength, and range of motion was compared pre- and post-rehabilitation using analysis of covariance (covariates: age, gender). Exercise-induced changes in MRI, and patient functional outcome measures were correlated using Pearson’s correlation test.

**Results:**

No significant differences were found in muscle size or fatty infiltration of the lumbar extensors over the course of rehabilitation (*p* > 0.31). However, patients reported reduced pain (*p* = 0.002) and were stronger (*p* = 0.03) at the conclusion of the program. Improvements in muscle size and quality for both multifidus and erector spinae correlated with improvements in disability, anxiety/depression, and strength.

**Conclusion:**

While average muscle size and fatty infiltration levels did not change with high-intensity exercise, the results suggest that a subgroup of patients who demonstrate improvements in muscle health demonstrate the largest functional improvements. Future research is needed to identify which patients are most likely to respond to this type of treatment.

## Background

Low back pain (LBP) is a debilitating condition, and is highly prevalent in the United States, affecting 65–85% of the population during their lifetime [1, 2]. Although acute LBP is thought to be self-limiting, recurrence and progression to chronic LBP is common, even when early treatment is sought [3]. Improving strength and stability of the trunk musculature through therapeutic exercise is a common physical rehabilitation goal for patients with LBP [4–7]. Success rates of therapeutic strategies vary, likely due to the high variability in exercise protocols and dosing prescriptions [7]. As such, there is a significant need for standardized dosing in studies involving exercise-based rehabilitation and controlled trials demonstrating differences in pain and functional outcomes after these interventions [8, 9].

Lumbar muscular atrophy and fatty infiltration (a measure of muscle quality) is closely correlated with LBP [10–13]. The lumbar extensors, which are thought to provide muscular stability to the vertebral column in order to prevent injury [14, 15], undergo accelerated atrophy and fatty infiltration in individuals with LBP as compared to age-matched healthy counterparts [10, 11]. Therefore, physiologic changes in muscle, such as hypertrophy and reversal of fatty infiltration, should be considered when assessing the effectiveness of physical rehabilitation. However, when comparing therapeutic strategies to target increasing trunk muscular strength, evidence is conflicting as to whether currently utilized exercise doses or modalities are sufficient to elicit a physiologic response of the muscle in the form of hypertrophy or reduction in fatty infiltration in the presence of LBP. In fact, changes in muscle size and reversal of fatty infiltration in this population are rarely observed in response to most exercise programs [3, 16–18]. The only studies reporting consistent increases in muscle cross sectional area (mCSA) utilize high intensity strengthening protocols [16, 19–22]. However, these studies did not assess whether high intensity strengthening protocols can reverse lumbar fatty infiltration.

Given the potential benefit of high-intensity rehabilitation, which may activate the lumbar extensor muscle groups to a degree required for muscle hypertrophy, successful implementation could result in better outcomes for patients with LBP. Therefore, the goal of this preliminary study was to evaluate mCSA and fatty infiltration in patients with LBP before and after undergoing a standardized, high-intensity, resistance-based exercise program. A secondary goal of this study was to correlate changes in mCSA or fatty infiltration with psychosocial and functional changes before and after rehabilitation. We hypothesized that mCSA of the lumbar muscles would increase and fatty infiltration would decrease in patients with LBP over the course of high-intensity, resistance-based physical rehabilitation. Additionally, we hypothesized that improvements in psychosocial and functional outcome measures over the course of rehabilitation would positively correlate with increases in mCSA and decreased fatty infiltration in the lumbar musculature.

## Methods

### Participants

The University of California, San Diego Institutional Review Board approved this study. All subjects provided oral and written consent to participate. Patients were recruited from an outpatient rehabilitation center, for which they were undergoing a standardized 10-week, high-intensity, resistance-based physical therapy program targeting increasing lumbar extensor strength as part of their prescribed care for a diagnosis of degenerative disc disease, stenosis, spondylosis, or nonspecific low back pain. After completion of the standard rehabilitation program, patients were recruited for this study if they had undergone a pre-treatment magnetic resonance imaging (MRI) scan as part of their prescribed care, were 18 or over, and had completed the entire rehabilitation protocol (20 visits). All patients were undergoing conservative care for their LBP symptoms. Patients did not receive financial compensation for participation in this study. Patients were included regardless of prior exercise experience or comorbidity. Patients who were not cleared by their physician to initiate an exercise rehabilitation program due to fracture or other spine pathology considered to be a contraindication to exercise or range of motion were not eligible for screening or recruitment. Patients were excluded if they had previously undergone surgery for a LBP related injury, or if they did not have a pre-rehabilitation MRI for comparison. An a priori power analysis to determine sample size was not performed. Rather, a convenience sampling approach was used to enroll eligible patients within the study timeframe (August 2016 – March 2018).

### Resistance-based exercise protocol

**Fig. 1:**
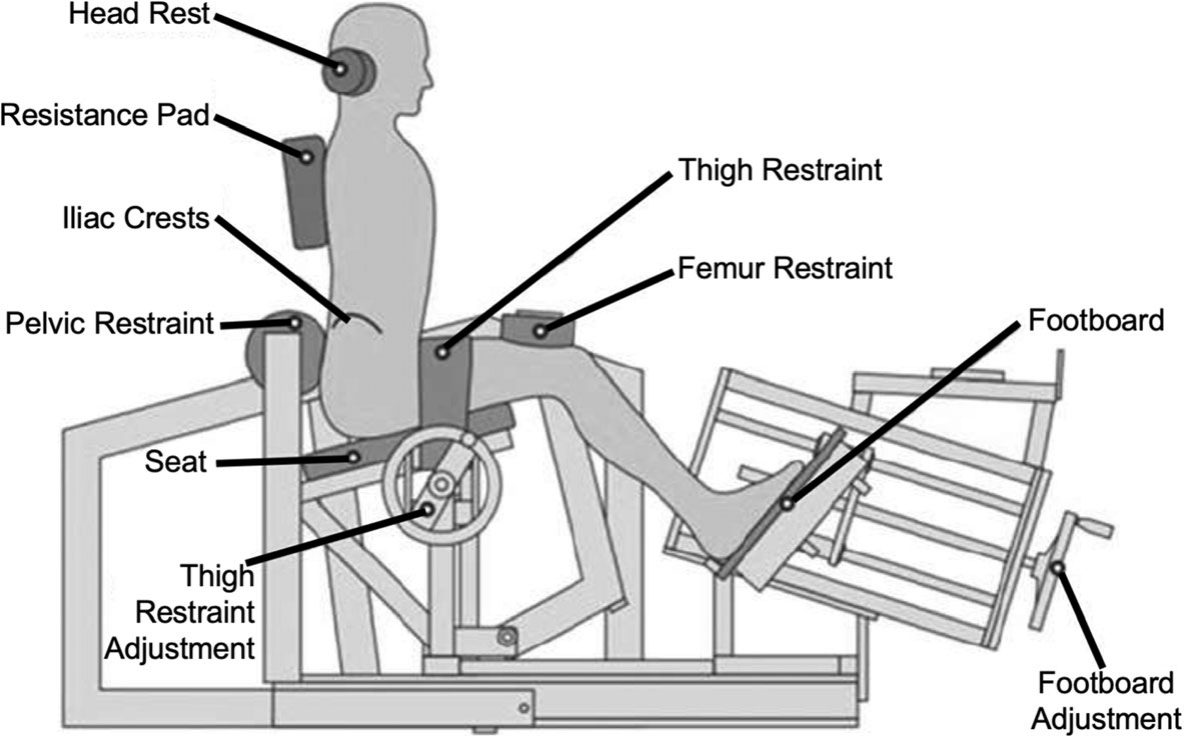
Schematic of the MedX isokinetic dynamometer. A patient is secured to the machine using pelvic, thigh, and femur restraints, and presses backwards against a resistance pad. This configuration isolates the lumbar musculature in extension. Image adapted from Fisher et al. with permissions [23]

Upon initiating the program, a physical examination was performed including measurements of lumbar strength and range of motion (ROM) using an isokinetic dynamometer (MedX Holdings Inc., Cheyenne WI; Fig. 1). This device allows for isolation of the lumbar extensors through pelvic stabilization in conjunction with measurements of torque across a monitored patient-tolerated range of motion. The patients were then individually prescribed lumbar extension resistance exercises on the MedX machine based on a maximal voluntary contraction. The exercises were performed throughout the maximum available range of motion that a patient was able to perform under supervision of a trained physical therapist. Each patient was assigned to a single physical therapist who supervised all of their training sessions for the duration of the program. Treatment exercise doses were prescribed at 60–80% of that maximal effort for 15–20 repetitions [24, 25]. Patients were instructed by the physical therapist to perform exercises throughout their available range of motion unless their symptoms increased with the exercise. Exercise was advanced in subsequent visits by 5–10% of the exercise load once they were able to tolerate > 20 repetitions without an increase in pain. If they were able to reach > 10 repetitions but < 20 repetitions, their exercise load remained the same at their next visit. If they were unable to reach 10 repetitions, their exercise load was decreased 5–10% at their next visit. Strength and ROM were measured from the machine-based torque measurements during lumbar extension exercises at each visit. Twenty visits over ten weeks was considered the standard regimen to complete the rehabilitation protocol. All patients were also provided with a copy of the, “Take Back Control” book upon initiation of care, which provides guidance on healthy lifestyle modifications such as remaining active and maintaining a healthy diet [26]. Any adverse event such as an increase in symptoms in response to treatment was reported to the treating physician for follow up.

### Image acquisition

Prior to rehabilitation, all subjects received pre-rehabilitation MRIs at an outpatient imaging facility as part of their medical care. This resulted in different magnet strengths and pulse sequence parameters used for each patient. In order to standardize musculoskeletal measurements across patients acquired at different facilities, T1-weighted images were used for all analysis.

Upon completing the rehabilitation protocol, post-rehabilitation MRIs of the lumbar spine (L1-S1) were acquired using a 3 T scanner (Discovery 750; GE Healthcare, Waukesha, WI) and a cardiac coil. The imaging protocol consisted of 1) a localizer scan and 2) an axial T1-weighted scan. The localizer was a fast spoiled-gradient echo with the following scanning parameters: TR, 5 milliseconds; TE, 2.3 milliseconds; FoV, 32 cm; acquisition matrix, 512 × 512; pixel size, 0.625 × 0.625 mm^2^; slice thickness, 1 mm; no gap; number of averages, 3. The axial T1-weighted scan was a fast spin echo with the following scanning parameters: TR, 849 milliseconds; TE, 12.3 milliseconds; FoV, 25.6 cm; acquisition matrix, 512 × 512; pixel size, 0.5 × 0.5 mm^2^; slice thickness, 4 mm; no gap; number of averages, 1.

### Muscle physiology measurements

**Fig. 2:**
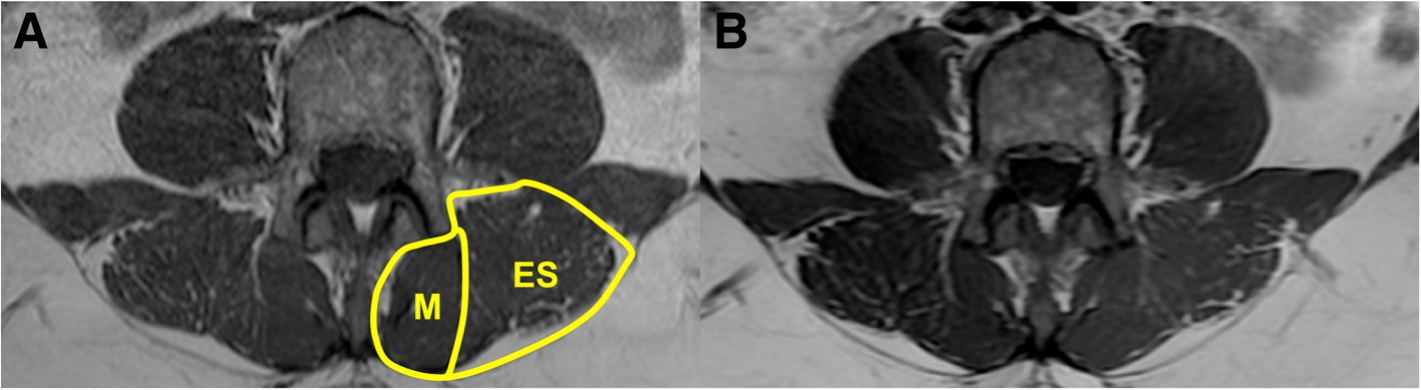
Example pre- (**a**) and post-rehabilitation (**b**) MR images of the lumbar musculature. The erector spinae (ES) and multifidus (M) muscles have been manually defined for the left side of the patient in A

Regions of interest (ROI) from T1-weighted axial MRIs were manually drawn around the multifidus and erector spinae (ES) muscles on a single slice estimated to be closest to the midlevel of the L4 vertebrae (Fig. 2) by an investigator blinded to patient group (pre- or post-rehabilitation) using OsiriX [27]. This method has been previously described, and has an inter-rater agreement > 0.928 [28]. If the plane of post-rehabilitation MRI’s were rotated compared to pre-rehabilitation MRI’s, post-rehabilitation MRI’s were registered to match the pre-rehabilitation MRI using the multi-planar reconstruction tool in OsiriX by a separate investigator. Similar registration techniques for MRI scans taken on separate days have been used and found excellent agreement between ROI based measures [29].

Muscle cross sectional area (mCSA) and fat fraction (FF; a measure of fatty infiltration) were measured within each ROI with custom written Matlab (Mathworks, Natick MA) software, which has been previously described [30]. Briefly, pixels were identified as muscle or fat by fitting a two term Gaussian model to the histogram of pixel intensities from within the segmented ROIs. The intersection of the two Gaussian curves was considered the threshold; pixel intensities above the threshold were classified as fat, pixel values below the threshold were classified as muscle. FF was calculated from the following equation:

$$ FF=\frac{\#_{pixels, fat}}{\#_{pixels, fat}+{\#}_{pixels, muscle}} $$

mCSA was calculated from the total cross-sectional area (tCSA) of the ROI [28]:

$$ mCSA= tCSA\ast \left(1- FF\right) $$

FF and mCSA data were averaged across sides for all subsequent analyses.

### Functional outcome measures

A 100 mm visual analog scale (VAS) was used to assess a patient’s perceived LBP at the beginning and end of rehabilitation, with higher values indicating more pain [31–33]. LBP related disability was assessed using the Oswestry Disability Index (ODI) questionnaire at the beginning and end of the program [34]. The Patient Health Questionnaire-4 (PHQ4) was used to assess patient depression/anxiety at the beginning and end of the program [35, 36]. Maximum lumbar extension strength was measured on the MedX isokinetic dynamometer at the beginning and end of the program, and was monitored throughout the program for verification of exercise intensity. ROM was measured in degrees as the maximum range of motion through which the patient was able to perform the resistance exercise.

### Statistical analysis

Demographic measures collected for each patient included gender, age, and weight. The primary outcome measures of muscle health were mCSA and FF. The primary functional outcome measures from this study were VAS, ODI, PHQ4, strength, and ROM. Normality of all variables was checked using a Shapiro-Wilk test. Each outcome measure was compared using a 1-way analysis of covariance (covariates: age, gender), to identify the effect of treatment on each outcome measure (factor: treatment). Pearson’s correlation test for normally distributed data or Spearman rank correlation test for non-normally distributed data was used to determine the strength of association between changes in muscle health, and functional (Strength, VAS, ODI) or psychosocial (PHQ4) improvements with the program. Statistical analysis was performed using SPSS version 25.0 (IBM Corporation, Armonk NY).

## Results

### Demographics

Fourteen patients volunteered for this study (Table 1). The majority of patients (*N* = 13) participating in this study were being seen for a primary diagnosis of degenerative disc disease, with secondary diagnoses of stenosis (*N* = 8) or spondylosis (*N* = 2). One subject was diagnosed as having nonspecific LBP. As a group, on average a 1.1 kg loss in weight was observed over the course of rehabilitation (*p* = 0.711).

**Table 1:** Mean ± standard deviation of the demographic measures of patients included in this study

*N* = 14	Pre-rehab	Post-Rehab
Age (years)	52.8 ± 14.8	
Gender (M:F)	7:7	
Weight (kg)	82.4 ± 15.81	81.3 ± 14.3
Visual Analog Scale (mm)	47.9 ± 22.2	20.7 ± 18.2
Strength (Nm)	196.8 ± 78.7	283.7 ± 131.9
ODI (%)	28.7 ± 12.4	26.4 ± 16.5
PHQ4	2.9 ± 5.0	1.9 ± 3.2
Range of Motion (°)	61.0 ± 10.9	66.0 ± 8.2

*M* male, *F* female, *kg* kilogram, *ODI* Oswestry Disability Index, *PHQ4* Patient Health Questionaire-4

### Effect of high-intensity resistance exercise on MRI-based measures of muscle health

**Fig. 3:**
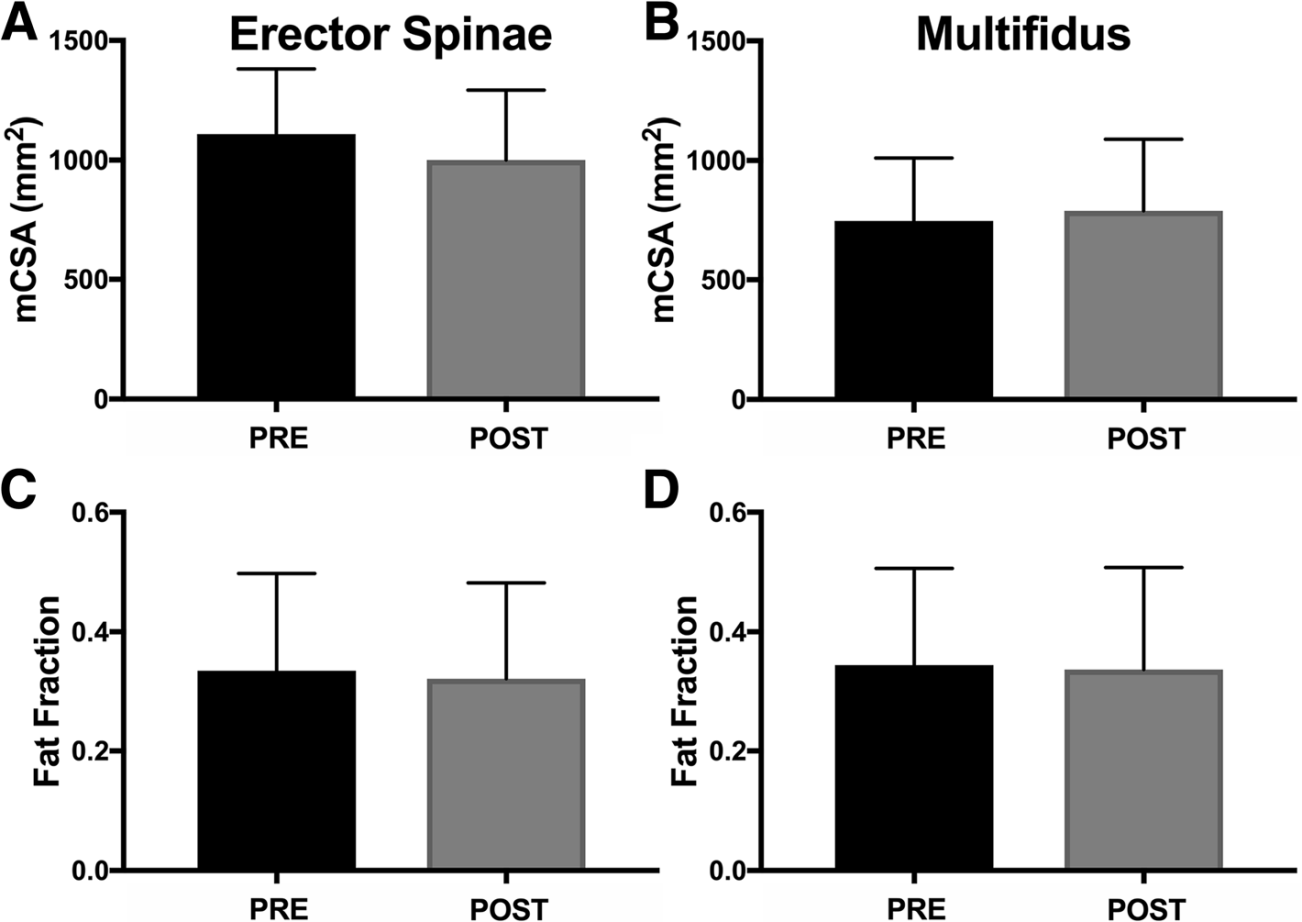
Muscle cross sectional area (mCSA) (**a**, **b**) and fat fraction (FF) (**c**, **d**) measures of the erector spinae (**a**, **c**) and multifidus (**b**, **d**) muscles. No significant differences in muscle physiology assessed by magnetic resonance imaging were found between pre- and post-rehabilitation measurements. Data reported as mean ± standard deviation

Small changes were observed for mCSA of the erector spinae (− 7.9 mm^2^; F(1,23) = 0.063; *p* = 0.804) and multifidus (+ 41.6 mm^2^; F(1,23) = 0.026; *p* = 0.873) when controlling for age and gender, which were likely to be observed by chance (Fig. 3A, B). Additionally, small changes were observed for FF of the erector spinae (− 0.013; F(1,23) = 1.079; *p* = 0.310) and multifidus (− 0.007; F(1,23) = 0.331; *p* = 0.570) when controlling for age and gender, which were also likely to be observed by chance (Fig. 3C, D). The covariate of age predicted FF for both muscles and mCSA for only multifidus (*p* < 0.01); older patients had smaller mCSA and higher FF. Males had a greater multifidus mCSA (*p* = 0.008).

### Effect of high-intensity resistance exercise on functional outcomes

**Fig. 4:**
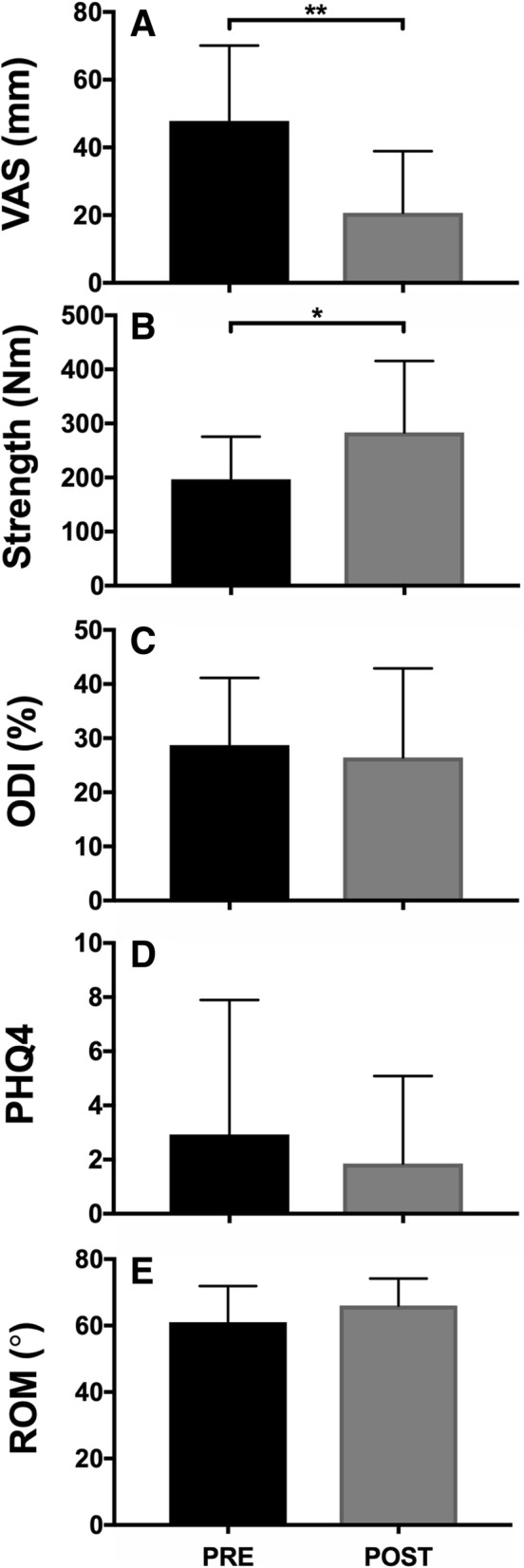
Functional outcome measures pre- and post-rehabilitation. VAS (**a**) was measured to assess pain. Strength (**b**) and ROM (**e**) were measured using a MedX isometric dynamometer. ODI (**c**) was used to assess low back pain related disability. PHQ4 (**d**) was used to assess anxiety/depression related to low back pain. * indicates *p* < 0.05. ** indicates *p* < 0.01. Data reported as mean ± standard deviation

On a group level, a 27.2 mm decrease in pain (*p* = 0.002), a 86.9 Nm increase in strength (*p* = 0.03) was observed (Table 1; Fig. 4A,B) . Additionally, a 2.3% decrease in ODI assessed disability (*p* = 0.689), a 1.0 point decrease in PHQ4 assessed anxiety/depression (*p* = 0.518) and a 5.0 ° increase in ROM (*p* = 0.173) was observed over the course of treatment (Table 1; Fig. 4C-E). At the conclusion of the standard rehabilitation period (20 visits), 7 patients decided to continue with a high-intensity, resistance based maintenance exercise program.

### Relationships between functional outcomes and MRI-based measures of muscle health

**Fig. 5:**
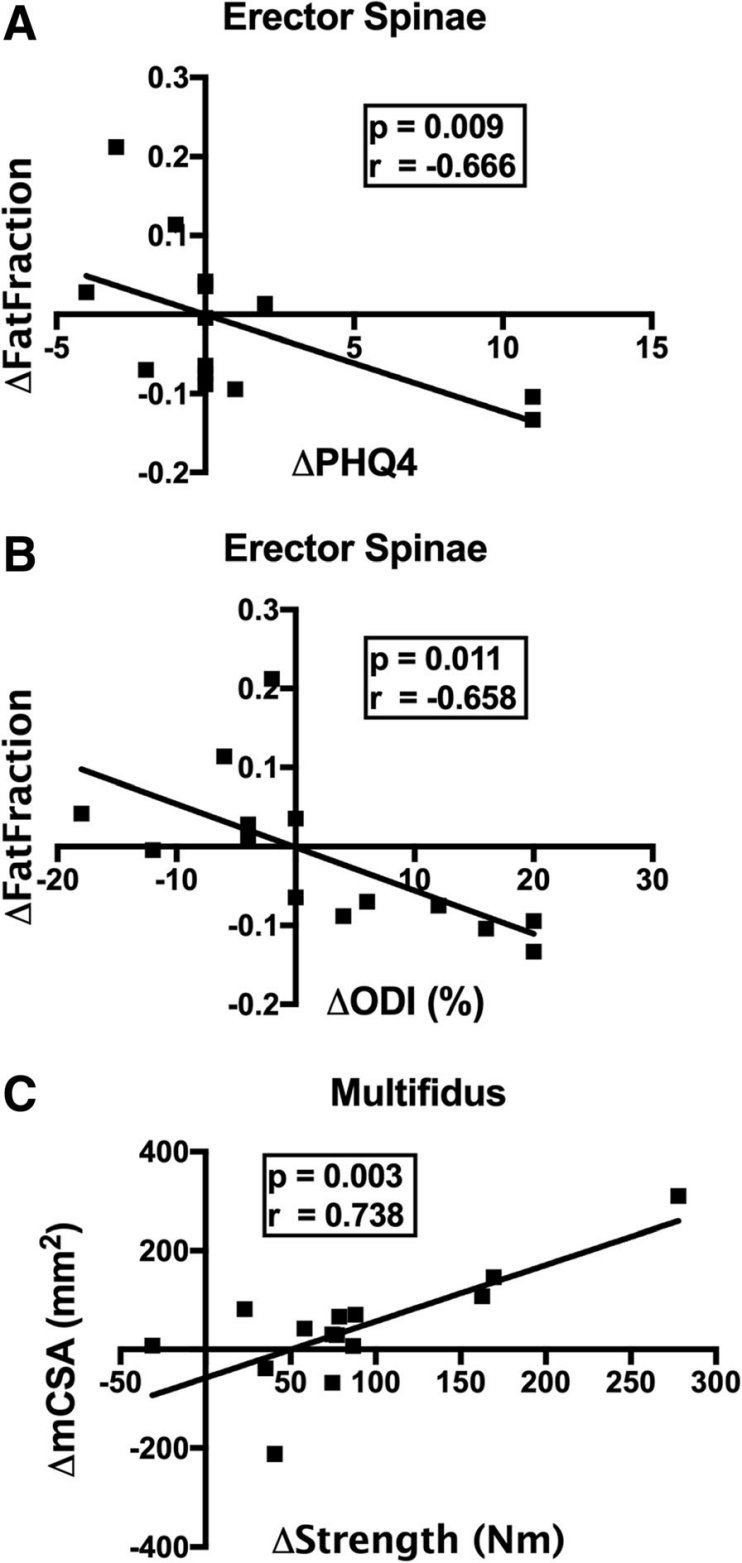
Significant correlations between patient reported outcome measures (x-axis) and muscle health measured with MRI (y-axis)

All functional outcome and MRI-based measures of muscle health were normally distributed except for PHQ4 (*p* < 0.001) and ROM (*p* = 0.005). Correlations were observed between improvements in functional outcomes and both FF and mCSA. Reductions in ES FF were associated with decreased depression/anxiety with the program (*p* = 0.009; r = − 0.666; Fig. 5A) as well as decreased LBP related disability (*p* = 0.011; r = − 0.658; Fig. 5B). Larger improvements in multifidus mCSA in response to the program were associated with increased isometric lumbar strength (*p* = 0.003; r = 0.738; Fig. 5C). However, the observed change in strength was not found to correlate with the change in pain (r = − 0.253; *p* = 0.382), disability (r = 0.345; *p* = 0.228), or depression/anxiety (r = − 0.329; *p* = 0.251) over the course of rehabilitation.

## Discussion

This was a preliminary study, evaluating changes in mCSA and FF in response to a standardized, high-intensity, machine-based resistance exercise program in patients with LBP. We hypothesized that increased mCSA and decreased FF would be observed after this program in conjunction with improvements in patient functional outcomes. These data demonstrate that on average, patients did not demonstrate improvements in muscle size or quality as measured by T1-weighted MRI in response to this program, despite improvements in strength and reduced pain. Correlations were found between functional and MRI measured outcomes, in that patients who demonstrated improvements in muscle health also demonstrated the largest functional improvements in LBP related disability, strength, and depression/anxiety. These findings, although limited by a small sample size, suggest that while resistance based exercise may not result in improvement in muscle health and functional improvements for all patients with LBP, there are some patients who demonstrate the largest improvements in muscle health and are also the most responsive to treatment and vice versa.

The magnitude of change in muscle health and functional outcomes is similar to prior studies on the effects of exercise on individuals with LBP for pain-specific outcomes, but not for outcomes of disability or depression/anxiety [37–39]. The cohort’s average pain reduction was 27.1 mm ± 20.9 mm, which exceeds the minimal clinically important difference (MCID) of 20 mm for VAS in patients with LBP [37]. Previous exercise trials in these populations report improvements in pain ranging from 7 to 13 points, and reductions in LBP related disability of between 6.9 to 20 points [38, 39]. The smaller treatment effects for LBP related disability and anxiety/depression may be due to the low initial scores for levels of LBP related disability and depression/anxiety in this cohort as compared to other studies [37–39]. Interestingly, while the rehabilitation protocol made patients stronger, and muscle health was related to treatment-related strength gains, the amount of strength gained did not correlate directly with functional improvements.

The muscle-specific changes that have been reported in response to various exercise programs demonstrate conflicting results, and have largely been based on measures of muscle size. To our knowledge, only two other studies have investigated changes in multifidus fatty infiltration in response to an exercise program. One study only included males in their cohort, and found similar results using an isolated lumbar extension protocol with variable frequency [17]. A study by Welch et al. did find decreases in lumbar paraspinal muscle FF by 2.5% [40]. However their patient cohort was young with lower baseline FF, so their rehabilitation protocol may not exhibit muscle physiological changes in the average patient population with LBP. A systematic review by Shahtahmassebi et al. reported that only one of 7 exercise intervention studies reported significant improvements in multifidus size after a machine-based resistance exercise program [19, 41]. Even when these studies were expanded to include other exercise modalities such as motor control or aerobic exercises, only two of 18 studies reported improvements in multifidus CSA after the program [20]. Our study is consistent with the prior literature in that there is no strong evidence of a mean change in muscle size or quality. However, our data may provide preliminary evidence that the lack of an appreciable effect size in response to exercise when averaged across the whole study population may be because treatment outcomes are combined across responder and non-responder patient subgroups, whose distinguishing characteristics have yet to be identified or well-defined. When we further examined patients who demonstrated improvements in muscle health parameters, we found patients who had lower levels of baseline disability and anxiety/depression had larger improvements in muscle physiology for ES only, however no such trend was observed for multifidus. This suggests that patents with less anxiety/depression initially are more likely to experience significant physiologic changes from high-intensity, resistance based exercise.

There are several limitations to this study. First, as this was a preliminary study, our sample size was small. However, we observed similar results to a similar study with a larger sample size by Welch et al., highlighting the potential importance of identifying patient subgroups [40]. Additionally, as patients were only enrolled in this study if they had pre-rehabilitation MR imaging, the pre-rehabilitation MRIs were acquired at outside facilities. This resulted in different MR field strengths, MR acquisition parameters, and patient positioning in the scanner. We attempted to minimize error by measuring the spine at the same location (L4), in both pre- and post-rehabilitation images. The L4 level was chosen as L4 fat signal fraction correlates highly (R^2^ > 0.92) with whole lumbar fat fraction [42]. Furthermore, we registered post-rehabilitation MR images to precisely match slice orientations across acquisitions in 3D, in order to ensure that the same area of the muscles was being analyzed in both the pre- and post-rehabilitation images. Standard T1-weighted MRI of the lumbar musculature may be too crude a technique to assess early muscle adaptation to resistance based training, as it only has the ability to detect changes on a whole muscle level. Chemical shift imaging techniques such as Dixon or IDEAL MRI allow for intravoxel quantification of fat, and may be more sensitive to changes in the muscle fat content [43, 44]. Furthermore, diffusion tensor imaging (DTI) - a MRI based technique sensitive to muscle microstructure - may provide more sensitivity to changes in muscle microstructure associated with exercise such as fiber hypertrophy. Previous studies have shown differences in DTI derived measures before and after strenuous exercise that were not detected with routine T1- and T2-weighted MRI [45, 46]. Therefore, future studies may consider incorporating chemical shift imaging or DTI based techniques to better assess microstructural changes associated with resistance-based exercise in patients with LBP.

In addition to registering pre- and post-rehabilitation images, we took several additional steps to minimize bias and error in this study. We attempted to minimize bias introduced with manual thresholding techniques by using an automated technique to determine the fat/muscle pixel intensity threshold. Additionally, due to the fact that this study employed convenience sampling, we were unable to control for patient-specific factors such as LBP duration, method of injury, previous treatments, etc. Without controlling for these characteristics, it is more difficult to understand which population may benefit most from this resistance based physical rehabilitation program. A future prospective study with a well-characterized patient population could address these issues.

A unique feature of this study was the standardization of the exercise dosing across all patients. While resistance based exercise is generally associated with good outcomes, dosing and protocol variations make it difficult to compare across studies. The rehabilitation exercise protocol used in this study was found to significantly reduce pain and increase strength across patients. Most importantly, we observed that patients who demonstrate changes in muscle physiology respond better to a high-intensity resistance based exercise program, which must be more closely investigated in future experiments.

This exercise program followed in this study was developed by the SpineZone Rehabilitation Center (San Diego, CA). The exercise protocol and progression as based on prior literature using machine-based resistance exercise to strengthen the posterior lumbar musculature, as well as modifications made by trained physical therapists to accommodate individuals with pain and pathology for safety in concordance with the ACSM guidelines for older or more fragile populations [24, 47–49]. A single set was utilized because in prior research on lumbar extension-specific exercise, it was demonstrated to induce the largest changes at a 2x per week frequency when compared to a higher number of sets [47]. Although the modifications used in this study have not been rigorously studied in the literature, it was found that in order for patients to achieve a perceived exertion of greater than 7 without exacerbation of symptoms, a higher number of repetitions at a submaximal intensity was required. We recognize this as a potential limitation, however this program aimed to optimize strength training with symptom reduction without risking patient safety.

## Conclusions

In this study, we assessed mCSA and FF of the lumbar paraspinal muscles over the course of high-intensity resistance rehabilitation in patients with LBP. Although only slight group wide changes in average muscle size or fatty infiltration were observed, overall patients experienced large improvements in pain and strength. Improvements in disability, anxiety/depression, and strength were found to correlate with increased mCSA and decreased FF, which suggests that high-intensity, resistance exercise elicits a physiologic response in the lumbar muscles of a subgroup patients with LBP. These preliminary findings may suggest that there are some patients who experience a more robust response to this intervention strategy, although the sample size of this study precludes this conclusion. Future research is needed to identify whether these subgroups exist, and which patients may benefit most from this rehabilitation approach.

## Data Availability

The datasets used and/or analyzed during the current study are available from the corresponding author on reasonable request.

## Abbreviations

ES: Erector Spinae
FF: Fat fraction
LBP: Low back pain
MCID: Minimal clinically important difference
mCSA: Muscle cross sectional area
MRI: Magnetic resonance imaging
ODI: Oswestry disability index
PHQ4: Patient health questionnaire – 4
ROI: Region of interest
ROM: Range of motion
VAS: visual analog score
